# Iodine-131 treatment of thyroid cancer cells leads to suppression of cell proliferation followed by induction of cell apoptosis and cell cycle arrest by regulation of B-cell translocation gene 2-mediated JNK/NF-κB pathways

**DOI:** 10.1590/1414-431X20165933

**Published:** 2017-01-16

**Authors:** L.M. Zhao, A.X. Pang

**Affiliations:** 1 Department of Nuclear Medicine, Linyi People's Hospital, Linyi, China; 2 Department of Urology, Linyi People's Hospital, Linyi, China

**Keywords:** Iodine-131, P53, BTG2, SW579, Thyroid cancer, JNK/NF-κB pathways

## Abstract

Iodine-131 (^131^I) is widely used for the treatment of thyroid-related diseases. This study aimed to investigate the expression of *p53* and *BTG2* genes following ^131^I therapy in thyroid cancer cell line SW579 and the possible underlying mechanism. SW579 human thyroid squamous carcinoma cells were cultured and treated with ^131^I. They were then assessed for ^131^I uptake, cell viability, apoptosis, cell cycle arrest, p53 expression, and *BTG2* gene expression. SW579 cells were transfected with BTG2 siRNA, p53 siRNA and siNC and were then examined for the same aforementioned parameters. When treated with a JNK inhibitor of SP600125 and ^131^I or with a NF-κB inhibitor of BMS-345541 and ^131^I, non-transfected SW579 cells were assessed in JNK/NFκB pathways. It was observed that ^131^I significantly inhibited cell proliferation, promoted cell apoptosis and cell cycle arrest. Both BTG2 and p53 expression were enhanced in a dose-dependent manner. An increase in cell viability by up-regulation in *Bcl2* gene, a decrease in apoptosis by enhanced *CDK2* gene expression and a decrease in cell cycle arrest at G_0_/G_1_ phase were also observed in SW579 cell lines transfected with silenced *BTG2* gene. When treated with SP600125 and ^131^I, the non-transfected SW579 cell lines significantly inhibited JNK pathway, NF-κB pathway and the expression of BTG2. However, when treated with BMS-345541 and ^131^I, only the NF-κB pathway was suppressed. ^131^I suppressed cell proliferation, induced cell apoptosis, and promoted cell cycle arrest of thyroid cancer cells by up-regulating B-cell translocation gene 2-mediated activation of JNK/NF-κB pathways.

## Introduction

The history of radionuclide therapy for the treatment of various diseases dates back to early 1900's. A parameter considered while choosing a particular radionuclide for therapy is the effective half-life, which is the net half-life considering both physical and biological half-life within the patient's body or organs. The biological half-life of a radionuclide depends on parameters like radiotracer delivery, uptake, metabolism, clearance, and excretion within the patient's body. The ionizing radiation leads to DNA damage, which is primarily caused by both direct or indirect interaction of radiation leading to molecular damage such as single strand break, double-strand breaks, base damage and DNA-protein cross links (1–4). It is established that cancer cells are more prone to damage following exposure to ionizing radiation than normal cells, which leads to the death of cancerous cells (5). The most widely used therapeutic radionuclide for the treatment of thyroid-related diseases such as differentiated thyroid cancer, Grave's disease, solitary hyper-functioning nodule, and toxic multinodular goiter is iodine-131 (^131^I). ^131^I, an isotope of ^127^I, is commonly used as a beta emitter in radiation therapy, causing mutation and cell death. It is known that 10% of the energy and radiation dose is via gamma radiation. In a study by Eriksson et al. (6), radio-immunotherapy triggered apoptosis in tumor cells.

Expression of p53 at post-translational level is enhanced due to DNA damage by radiation (7), subsequently leading to the arrest of cell growth at G1 and/or G2 phase, DNA repair, senescence or apoptosis (7–10). B-cell translocation gene 2 (BTG2) acts as a tumor suppressor gene for a number of cancers and it is stimulated by a p53-dependent pathway, which subsequently leads to the DNA damage. *BTG2* gene belongs to an anti-proliferative family protein which has highly conserved domains of BTG-Box A (Y50–N71) and BTG-Box B (L97–E115) (11–14). It has been reported that amongst the numerous molecules that are involved in diverse anti- or pro-apoptotic signaling pathways, NF-kB is one of the key factors controlling anti-apoptotic responses. The anti-apoptotic effect is thought to be mediated through not only transcriptional activation of dependent genes but also by cross talking with the JNK pathway (15). In the present study, we have assessed the effects of ^131^I in thyroid cancer cell line SW579 with special emphasis on cell proliferation, apoptosis, and cell cycle arrest, and also explored the possible underlying mechanisms in JNK/NF-kB pathways.

## Material and Methods

### Cell culture

SW579 human thyroid squamous cell carcinoma cells were obtained from American Type Culture Collection (USA), and cultured in L-15 medium (GE Healthcare Life Sciences, USA) supplemented with 10% fetal calf serum (Gibco, USA), 2 mM glutamine (Gibco), penicillin (100 U/mL; Sigma-Aldrich, USA) and streptomycin (100 μg/mL; Amresco, USA), and maintained at 37°C without CO_2_ in a humidified atmosphere. SP600125 (10 μM) and BMS-345541 (10 μM) were used as JNK and NF-κB inhibitors to treat SW579 for 3 days, respectively (16).

### ^131^I uptake assay

The cells were seeded at 1×10^5^/well on 6-well plates for 24 h. Subsequently, the cells were cultured for 24 h with 2 mL culture medium per well containing 7.4, 14.8, 29.4 MBq/mL ^131^I (9).

### CCK-8 assay

SW579 cells were seeded on 96-well plate with 5000 cells/well, and cell proliferation was assessed by the Cell Counting Kit-8 (CCK-8, Dojindo Molecular Technologies, USA). Briefly, after stimulation, the CCK-8 solution was added to the culture medium, and the cultures were incubated for 1 h at 37°C in humidified 95% air and 5% CO_2_. The absorbance was measured at 450 nm using a Microplate Reader (Bio-Rad, USA).

### Apoptosis assay

Cell apoptosis analysis was performed using propidium iodide (PI) and fluorescein isothiocynate (FITC)-conjugated Annexin V staining. Briefly, cells were washed in phosphate-buffered saline (PBS) and fixed in 70% ethanol. Fixed cells were then washed twice in PBS and stained in PI/FITC-Annexin V in the presence of 50 μg/mL RNase A (Sigma-Aldrich), and then incubated for 1 h at room temperature in the dark. Flow cytometry analysis was done by using a FACScan (Beckman Coulter, USA). Data were analyzed with FlowJo software.

### Cell cycle assay

For analysis of cell cycle, cells with different treatments were trypsinized, washed twice in PBS, and fixed overnight at –20°C in 300 μL PBS and 700 μL ethanol. The fixed cells were spun down gently in 200 μL extraction buffer (0.1% Triton X-100, 45 mM Na_2_HPO_4_ and 2.5 mM sodium citrate) at 37°C for 20 min and then stained with PI (BD Biosciences, USA) (50 μg/mL) containing 50 μg/mL RNase A for 30 min at 37°C in the dark, and subsequently analyzed by FACScan. The experiment was repeated at least three times, and the data were analyzed using CellQuest and ModFit softwares (Verity Software House, USA).

### qRT-PCR

Total RNA was extracted with TRIzol reagent according to the manufacturer's protocol (Sigma) and 2 µg were reverse-transcribed with the Omniscript RT kit (Qiagen, Italy) using random primers (1 mM) at 37°C for 1 h. Real time PCR was performed in triplicate in 20 mL reaction volumes using the Power SYBER Green PCR Master Mix (Applied Biosystems, USA). All primers were purchased from Invitrogen Life Technologies (USA). Real time PCR reactions were carried out in a MJ MiniTM Personal Thermal Cycler apparatus (Bio-Rad Laboratories, USA). Melting curves were obtained by increasing the temperature from 60 to 95°C with a temperature transition rate of 0.5°C/s. The comparative threshold cycle number (CT) method was used to assess the relative quantification of gene expression. The fold change of the target gene was calculated as 2^-ΔΔCT^.

### siRNAs transfection

BTG2 siRNA, p53 siRNA, and siNC were designed and synthesized by GenePharma (China). Cell transfection was performed using Lipofectamine 3000 (Invitrogen Life Technologies) according to the manufacturer's instructions.

### Statistical analysis

All experiments were repeated three times. The results of multiple experiments are reported as means±SD. Statistical analyses were performed using SPSS 19.0 statistical software. Differences were compared using a one-way analysis of variance (ANOVA). A P-value of <0.05 was considered to be statistically significant.

## Results

### ^131^I inhibited cell proliferation, promoted cell apoptosis, and induced cell cycle arrest

**Figure 1:**
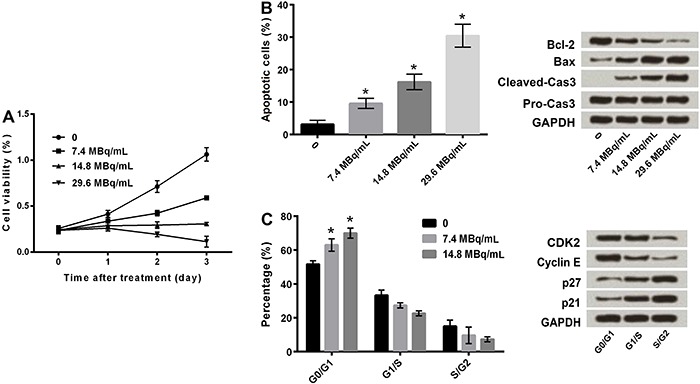
Effects of iodine-131 (^131^I) on cell proliferation (*A*), cell apoptosis (*B*) by regulating apoptosis-related protein, and cell cycle arrest (*C*) by modulating cell cycle-related protein. Data are reported as means±SD. *P<0.05 compared with control (CTL - GAPDH) (ANOVA).

^131^I was found to inhibit cell proliferation when administered to SW579 human thyroid squamous cell carcinoma cell lines. Cell viability was lesser than 0.5% at 14.8 (P<0.05) and 29.4 MBq/mL (significantly lower than cell viability at 7.4 MBq/mL; Figure 1A). A significant increase in apoptosis was observed when SW579 cells was treated with ^131^I at 29.6 and 14.8 MBq/mL (P<0.05; Figure 1B). Furthermore, expression of Bcl-2 was suppressed by 0.5 fold, and *Bax* and *cleaved-Cas 3* genes were enhanced by 1.5 and 1.5 folds, respectively, at 14.8 MBq/mL compared to GAPDH expression used as endogenous control. ^131^I induced cell cycle arrest significantly by more than 60% at G_ο_/G_1_ at the concentration of 14.8 MBq/mL compared to the arrest at G_1_/S and S/G_2_, by suppressing the expression of cyclin-dependent kinases 2 (CDK2) and cyclin E by 0.5 and 0.4 folds, respectively, at 14.8 MBq/mL compared to GAPDH expression (P<0.05). Furthermore, the expressions of *p27* and *p21* genes were enhanced by 1.5 and 2 folds, respectively, at 14.8 MBq/mL compared to GAPDH expression (Figure 1C).

### ^131^I induced the expressions of p53 and BTG2

**Figure 2:**
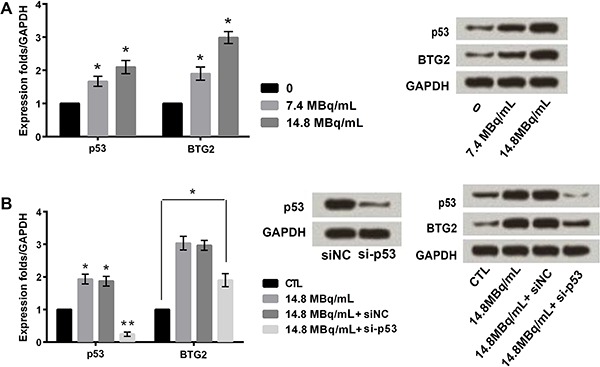
Effects of different concentrations of iodine-131 (^131^I) on expression of p53 and BTG2 (*A*). *B*, Expression of BTG2 was raised even with silencing of p53 (si-p53). Data are reported as means±SD. *P<0.05. **P<0.01 compared with control (CTL - GAPDH) (ANOVA).

As shown in Figure 2A, ^131^I increased the expressions of p53 and BTG2 in a concentration-depended manner. The expression of BTG2 was raised even after silencing of p53, thereby indicating that the higher expression of BTG2 was only partly dependent on p53 expression (P<0.05) (Figure 2B).

### Silencing of BTG2 reversed the effects of ^131^I on cell proliferation, cell apoptosis, and cell cycle arrest

**Figure 3:**
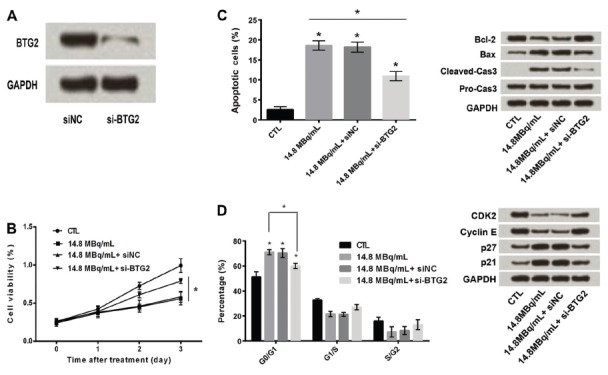
*A*, Transfection efficiency of BTG2. Silencing of BTG2 increased iodine-131 (^131^I)-induced cell proliferation (*B*), ^131^I-induced cell apoptosis (*C*), and down-regulated ^131^I-induced cell cycle arrest (*D*). Data are reported as means±SD. *P<0.05 compared with control (CTL - GAPDH) (ANOVA).

SW579 cells transfected with silenced *BTG2* gene (Figure 3A) and treated with ^131^I, presented an increase in cell viability (more than 0.5%) at 14.8 MBq/mL (Figure 3B), unlike in non-transfected cells, shown in Figure 1. Similarly, a significant decrease in apoptosis (approximately 10%) was found in cells transfected with silenced *BTG2* gene compared to non-transfected cells, where apoptosis was approximately 20%, when treated with ^131^I (Figure 3C). A down-regulation in Bcl2 and an up-regulation in Bax by 2.0 and 1.2 folds, respectively, were observed in cell proliferation pathway. A significant decrease in cell cycle arrest (less than 60%) was also observed at G_o_/G_1_ stage in cells transfected with silenced *BTG2* gene compared to non-transfected cells. Assessment of the molecular pathway revealed that there was an up-regulation in CDK2, followed by down-regulation in *cyclin E* and *p27* genes and down-regulation in *p21* gene (Figure 3D).

### ^131^I up-regulated BTG2 expression by activation of JNK/NF-κB pathways

**Figure 4:**
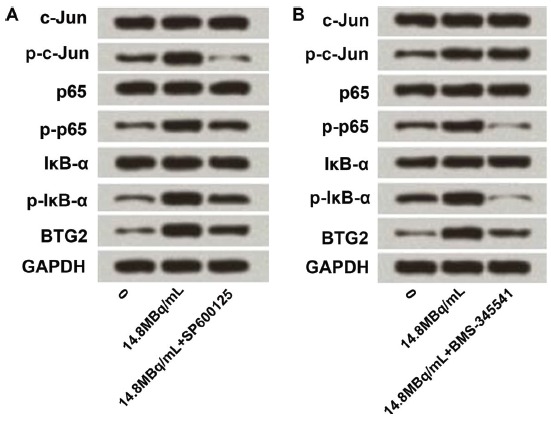
*A* and *B*, Iodine-131 up-regulated BTG2 expression by activation of JNK/NF-κB pathways.

As shown in Figure 4, non-transfected SW579 cells treated with SP600125, a JNK inhibitor, and ^131^I at 14.8 MBq/mL not only had a significant inhibition of JNK pathway but also of NF-κB pathway. The expression of BTG2 was also down-regulated. Furthermore, non-transfected SW579 cells treated with BMS-345541, a NF-κB inhibitor, and ^131^I at 14.8 MBq/mL had only the expression of NF-κB pathway affected but not of the JNK pathway. The expression of BTG2 was down-regulated, thus indicating that ^131^I up-regulated BTG2 expression by activation of JNK/NF-κB pathways.

## Discussion

It is well known that ^131^I destroys residual thyroid cancer tissue after surgical resection of differentiated thyroid carcinoma. The degree to which DNA is damaged by ionizing radiation depends on factors like type and dose of radiation (17,18). In the present study, we evaluated the role of ^131^I in cell proliferation, apoptosis and cell cycle arrest in a thyroid cancer cell line, together with the exploration of the possible underlying mechanism (increased expression of *BTG2* gene-mediated activation of the JNK/NF-κB pathways). ^131^I significantly inhibited cell proliferation as assessed in terms of cell-viability, enhanced cell apoptosis by down-regulating *Bcl2* gene, and promoted cell cycle arrest at G_0_/G_1_ phase by down-regulating *CDK2* gene. Cell apoptosis is largely regulated by protein-protein interactions between members of the Bcl-2 protein family. It is known that members of *Bcl-2* family genes have conserved domains called Bcl-2 homology domains, which are differentially modulated in various cancers (19,20).

Furthermore, ^131^I increased both BTG2 and p53 expression in a dose-dependent manner. It is mportant to mention that ^131^I enhanced the expression of BTG2, after silencing *p53* gene in SW579 cells, suggesting that the expression of BTG2 was partly dependent on the *p53* gene. An increase in cell viability by up-regulation in *Bcl2* gene, a decrease in apoptosis by enhanced *CDK2* gene expression and a decrease in cell cycle arrest at G_0_/G_1_ phase were also observed in SW579 cells transfected with silenced *BTG2* gene. Moreover, it was observed that not only the JNK pathway in the non-transfected SW579 cells, treated with SP600125, a JNK inhibitor, and ^131^I at 14.8 MBq/mL, was significantly inhibited but also the NF-κB pathway was inhibited along with the down-regulation of the BTG2 expression. Again, when treated with BMS-345541, a NF-κB inhibitor, and ^131^I, SW579 cells revealed only suppression of the NF-κB pathway but not that of the JNK pathway. Considering the aforementioned effects of ^131^I, we can conclude that ^131^I up-regulated BTG2 expression by activation of JNK/NF-κB pathways.
